# Chaos game representation dataset of SARS-CoV-2 genome

**DOI:** 10.1016/j.dib.2020.105618

**Published:** 2020-04-25

**Authors:** Raquel de M. Barbosa, Marcelo A.C. Fernandes

**Affiliations:** a MIT Department of Chemical Engineering, Massachusetts Institute of Technology, Cambridge, MA, 02142, USA; b Laboratory of Machine Learning and Intelligent Instrumentation, IMD/nPITI, Federal University of Rio Grande do Norte, Natal 59078-970, Brazil; c Department of Computer Engineering and Automation, Federal University of Rio Grande do Norte, Natal, RN, 59078-970, Brazil

**Keywords:** SARS-CoV-2, CGR, COVID-19

## Abstract

As of April 16, 2020, the novel coronavirus disease (called COVID-19) spread to more than 185 countries/regions with more than 142,000 deaths and more than 2,000,000 confirmed cases. In the bioinformatics area, one of the crucial points is the analysis of the virus nucleotide sequences using approaches such as data stream, digital signal processing, and machine learning techniques and algorithms. However, to make feasible this approach, it is necessary to transform the nucleotide sequences string to numerical values representation. Thus, the dataset provides a chaos game representation (CGR) of SARS-CoV-2 virus nucleotide sequences. The dataset provides the CGR of 100 instances of SARS-CoV-2 virus, 11540 instances of other viruses from the Virus-Host DB dataset, and three instances of Riboviria viruses from NCBI (Betacoronavirus RaTG13, bat-SL-CoVZC45, and bat-SL-CoVZXC21).

**Table:** 

Specification Table	
Subject	Biochemistry, Genetics and Molecular Biology (General)
Specific subject area	Bioinformatics
Type of data	Table
	Number
How data were acquired	NCBI - Genbank - SARS-CoV2 https://www.ncbi.nlm.nih.gov/genbank/sars-cov-2-seqs/
	Virus-Host-DB https://www.genome.jp/virushostdb/
	Matlab Software
	Excel Software
Data format	Raw and analyzed data are in Matlab file (.mat), Microsoft Excel file (.xlsx), and text file (.txt).
Parameters for data collection	The entire dataset was generated using MATLAB 2019b on Windows operating system with Intel Core - i5 6500T 2.5 GHz quad-core processor with 16GB of RAM.
Description of data collection	The raw data were downloaded from NCBI - Genbank, and Virus-Host-DB. The CGR values were generated using Matlab.
Data source location	Laboratory of Machine Learning and Intelligent Instrumentation, IMD/nPITI, Federal University of Rio Grande do Norte.
Data accessibility	https://data.mendeley.com/datasets/nvk5bf3m2f/1

## Value of the data

• These data are useful because they provide numeric representation of the COVID-2019 epidemic virus (SARS-CoV-2). With this form of the data, it is possible to use data stream, digital signal processing, and machine learning algorithms.
• All researchers in bioinformatics, computing science, and computing engineering field can benefit from these data because by using this numeric representation they can apply several techniques such as machine learning and digital signal processing in genomic information.
• Data experiments that use clustering and classification techniques in SARS-CoV-2 virus genomic information can be used with this dataset.
• These data represent an easy way to evaluate the SARS-CoV-2 virus genome.

## 1 Data Description

This work presents a new dataset of a chaos game representation (CGR) of SARS-CoV-2 virus nucleotide sequences. The dataset contains two kinds of data, the raw data, and the processing data. The raw data is composed of the 100 instances of the SARS-CoV-2 virus genome collected from the National Center for Biotechnology Information (NCBI) [1], 11540 instances of other viruses from the Virus-Host DB [2], [3], and three other instances of Riboviria also collected from the NCBI (Betacoronavirus RaTG13, bat-SL-CoVZC45, and bat-SL-CoVZXC21). Which have high similarity with SARS-CoV-2 [4], [5].

The dataset provides two groups of formats files for all data. In the first group, all data are stored in Matlab file format (.mat), and in the second group, part of the data is stored in Microsoft Excel (.xlsx) and another part in the text file (.txt). The two groups have the same information. The data is organized into three main directories: “SARS-CoV-2 data”, “Virus-Host DB data” and “Other viruses data.” Each main directory is formed by two sub-directories: “Matlab” and “Excel and txt.”

Each sub-directory “Matlab” contains three files called “RawDataTable.mat”, “RawData.mat” and “CGRData.mat”. “RawDataTable.mat” and “RawData.mat” files store the raw data information from the viruses database; they have the same information, however in the “RawDataTable.mat” the attributes are stored in Matlab table format (after 2013b version) and in “RawData.mat” the attributes are stored in Matlab cell arrays format. Each “CGRData.mat” file stores the CGR values of all viruses presented in each “RawDataTable.mat” and “RawData.mat” file. For the main directory “Virus-Host DB data”, the CGR values are stored in 10 files where each ***k***-th file is called “RawData_***k***.mat.”

Each sub-directory “Excel and txt” is composed of a file and another sub-directory called “RawData.xlsx” and “CGRData”, respectively. Each “RawData.xlsx” file has the raw data information from the viruses database, and each “CGRData” has the CGR of viruses presented in each “RawData.xlsx” file. The points of the CGR associated with each virus are stored in a text file called “LocusName_**COD**.txt” where **COD** is the code (locus name) associated with the virus in Genbank [6].

## 2 Experimental Design, Materials, and Methods

The Chaos Game Representation (CGR), proposed by H. Joel Jeffrey in [7], transforms the nucleotide sequence (DNA or RNA) to bi-dimensional real values. The CGR maintains the statistical properties of the nucleotide sequence, and it allows an investigation of the local and global patterns in sequences [8], [9].

The CGR has with input the nucleotide sequence, **s**, expressed as

(1) $$s=\left[s_{1},\cdots ,s_{n},\cdots ,s_{N}\right]$$

where *N* is the length of sequence and *s_n_* is the *n*-th nucleotide of the sequence. Each *n*-th nucleotide, *s_n_*, is mapped to bi-dimensional symbol (*s_x_*(*n*), *s_y_*(*n*)) and it can be expressed as

(2) $$s_{x}\left(n\right)=\left\{ \begin{array}{cc} 1 & \;\text{for}\;s_{n}=\text{A}\\ -1 & \;\text{for}\;s_{n}=\text{T}\;\text{or}\;\text{U}\\ -1 & \;\text{for}\;s_{n}=\text{C}\\ 1 & \;\text{for}\;s_{n}=\text{G} \end{array}\right.$$

and

(3) $$s_{y}\left(n\right)=\left\{ \begin{array}{cc} 1 & \;\text{for}\;s_{n}=\text{A}\\ 1 & \;\text{for}\;s_{n}=\text{T}\;\text{or}\;\text{U}\\ -1 & \;\text{for}\;s_{n}=\text{C}\\ -1 & \;\text{for}\;s_{n}=\text{G} \end{array}\right..$$

**Fig. 1:**
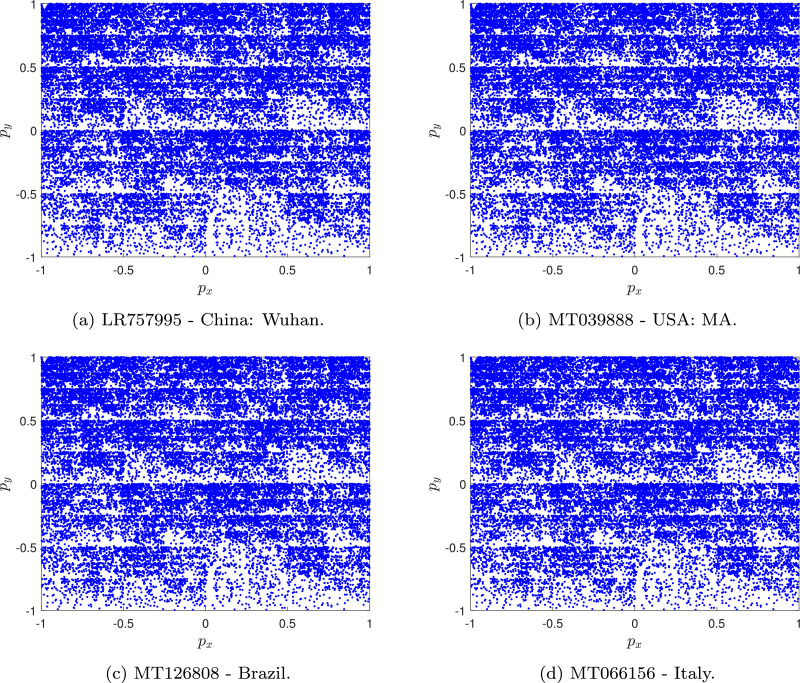
Example of the CGR values for the SARS-CoV-2 virus stored in this dataset.

After the mapping, each *n*-th symbol (*s_x_*(*n*), *s_y_*(*n*)) is transformed in CGR values by equations expressed as

(4) $$p_{x}\left(n\right)=\frac{1}{2}s_{x}\left(n\right)+\frac{1}{2}p_{x}\left(n-1\right)\text{,}\;\text{for}\;n=1,\cdots ,N$$

and

(5) $$p_{y}\left(n\right)=\frac{1}{2}s_{y}\left(n\right)+\frac{1}{2}p_{y}\left(n-1\right)\text{,}\;\text{for}\;n=1,\cdots ,N$$

where for the initial condition, n=0, $p_{x}\left(0\right)=\alpha_{x}$ and $p_{y}\left(0\right)=\alpha_{y}$ [7], [8]. The dataset was generated with $\alpha_{x}=0$ and $\alpha_{y}=0$. Figures 1(a), 1(b), 1(c) and 1(d) show a example of CGR points (*p_x_*(*n*), *p_y_*(*n*)) from dataset presented in this work.

## Appendix B Supplementary materials

Supplementary Data S1 Supplementary Raw Research Data. This is open data under the CC BY license http://creativecommons.org/licenses/by/4.0/ Supplementary Data S1
